# Relative Roles of TGF-***β*** and IGFBP-5 in Idiopathic Pulmonary Fibrosis

**DOI:** 10.1155/2011/517687

**Published:** 2011-01-26

**Authors:** A. Sureshbabu, E. Tonner, G. J. Allan, D. J. Flint

**Affiliations:** Strathclyde Institute of Pharmacy and Biomedical Sciences, University of Strathclyde, SIPBS Building, 161 Cathedral Street, Glasgow G4 0RE, UK

## Abstract

Although most evident in the skin, the process of scarring, or fibrosis, occurs in all major organs because of impaired epithelial self-renewal. No current therapy exists for Idiopathic pulmonary fibrosis. The major profibrotic factor is TGF-*β*1 and developing inhibitors is an area of active research. Recently, IGFBP-5 has also been identified as a profibrotic factor, and studies suggest that, while both TGF-*β*1 and IGFBP-5 activate mesenchymal cells to increase collagen and fibronectin production, their effects on epithelial cells are distinct. TGF-*β*1 induces cell death and/or EMT in the epithelial cells, exacerbating the disruption of tissue architecture. In contrast, IGFBP-5 induces epithelial cell spreading over collagen or fibronectin matrices, increases secretion of laminin, the epithelial basement membrane, and enhances the survival of epithelial cells in nutrient-poor conditions, as exists in scar tissue. Thus, IGFBP-5 may enhance repair and may be an important target for antifibrotic therapies.

## 1. Introduction

Idiopathic Pulmonary fibrosis (IPF) is a nonneoplastic chronic lung syndrome that is characterized by aberrant accumulation of fibroblasts/myofibroblasts and progressive abnormal remodelling of lung parenchyma, with subsequent scarring and disruption of its structure and function. It belongs to a broad category of 200 disorders called diffuse pulmonary lung diseases (DPLDs) or simply interstitial lung diseases (ILDs). This is further classified into a subgroup known as idiopathic interstitial pneumonia (IIP), where IPF is one of the seven diseases, being the most prevalent and pernicious disorder. It is subclassified with a pathological condition known as usual interstitial pneumonia (UIP). Major symptoms include chronic dyspnea (shortness of breath) induced by 6-minute walk test, persistent dry cough, reduced lung volume, and impaired gas exchange. Constitutional symptoms include weight loss, fatigue, finger clubbing, and general malaise [1]. IPF has a poor prognosis and is five times more prevalent than cystic fibrosis and amyotrophic lateral sclerosis, with no FDA approved treatments. Despite considerable recent progress, current treatment regimens are largely unpromising with a median survival rate of less than three years from the date of diagnosis [2]. The precise mechanisms involved in the pathogenesis of IPF have not been completely understood. Here, in this paper, we attempt to highlight the pathology of pulmonary fibrogenesis from its history, possible cellular and molecular mechanisms, and draw comparisons between the major profibrotic factor TGF-*β*1 and a more recently described player, IGFBP-5.

## 2. IPF Incidence and Pathophysiology

IPF is usually a disease of the elderly and occurs after five decades of life, with the probability of incidence markedly increasing thereafter. The onset of disease is slow but with age and time, the symptoms increase. There are five million sufferers, with increasing world-wide incidence, that are affected by IPF [3]. The number of affected patients has doubled in the past decade [4] and inevitably will increase in developed countries due to increasing age of the population. The disease is slightly more prevalent in men [5] and strongly associated with cigarette smoking [6]. Recent statistics of large United States and UK population-based studies indicated a significant increase in mortality [7, 8]. Annually, 40,000 and 4,000 new cases were diagnosed each year in US and UK, respectively.

Pulmonary fibrosis can also occur due to a variety of stimuli ranging from life style habits such as cigarette smoking [6], occupational exposure to polluted environments, notably asbestos or silica [9], induced by pharmacological agents [10], radiation exposure [11], and genetic predisposition [5], or associated with other autoimmune abnormalities such as collagen vascular diseases [12, 13].

## 3. Epithelial Cell Injury and IPF

The complex signalling mechanisms of pulmonary epithelial cell injury in response to fibrosis are of considerable research interest. As intact alveolar epithelium is critical for maintaining lung homeostasis, initiation of repair mechanisms following the insult and injury, without compromising its structure and function, is of great importance. Our understanding of the relationship between injury of epithelia and development of IPF has increased dramatically in recent years. Current hypotheses suggest that microrepetitive injury to pulmonary epithelial cells results in ineffective repair with subsequent fibrogenesis. As previously mentioned, the sources of recurrent injury could be wide ranging and include toxic or drug-induced insults, autoimmune disease, traumatic or hypoxic injuries, and bacterial or viral infections. Recently, a high occurrence of Epstein Barr Virus (EBV) expressing latent membrane protein (LMB-1) has been reported in IPF patients with synergizing effects with TGF-*β*-induced EMT in lung epithelial cells [14]. Observations suggest that the release of soluble growth factors after injury including chemokines, eicosanoids, and interleukins has also shown to be involved in the resolution of epithelial repair processes. However, there may be compromise with respect to structure and functional organisation with varying degree of injuries.

## 4. The Role of Fibroblasts/Myofibroblasts in IPF

Effective therapies to prevent tissue fibrosis require a complete understanding of the mechanisms involved in the development of the disease, and one mechanism of particular interest is fibroblast development. Resident fibroblasts and resident activated fibroblasts (myofibroblasts) are critical cell types for the process of wound healing and also for the formation of fibrotic lesions in the pathogenesis of lung fibrosis. Fibroblasts express receptors for a number of cytokines including PDGF [15], TGF-*β*1 [16], and TNF-*α* [16]. These cytokines and others may mediate their recruitment and activation during injury. In addition to their involvement in skin wound healing [17–19], myofibroblasts are most commonly identified and well characterized in idiopathic pulmonary fibrosis [20]. Myofibroblasts are mesenchymal cells with characteristics of both fibroblasts and smooth muscle cells [21]. The origin of myofibroblasts is still under debate. However, persistence of fibroblasts/myofibroblasts at the site of injury and extracellular matrix (ECM) are critical for fibrotic lesion formation in IPF [22]. Thus, understanding the mechanism of the gradual decrease of myofibroblasts, as occurs in normal wound healing, should be important for the resolution of pulmonary fibrosis.

## 5. Epithelial to Mesenchymal Transition

EMT is a prominent manifestation of cell plasticity observed in three distinct cellular processes: embryonic development, metastasis, and fibrosis. Here, as per the context, determination of the cellular source of myofibroblasts is crucial in understanding the pathogenesis of tissue fibrosis. Myofibroblasts appear to have at least three possible origins. Alveolar epithelial damage with consequent EMT, activation of fibroblasts, and circulating fibrocytes occur through detection of injurious signals including TGF-*β*, platelet-derived growth factor (PDGF), IL-13, and connective tissue growth factor (CTGF). This results in the transdifferentiation of myofibroblasts, with smooth muscle actin, MMP-2 and MMP-9 expression, and excessive collagen production [20]. Resident fibroblasts can respond to a variety of profibrotic mediators and differentiate into myofibroblasts [23]. TGF-*β*1 induces transdifferentiation of fibroblasts, through a Smad3-dependent mechanism, to myofibroblasts [24]. In vivo overexpression of IGFBP-5 induces increased expression of *α*-SMA and vimentin in dermal and lung fibroblasts suggesting fibroblast transdifferentiation [25, 26].

Epithelial-myofibroblast transdifferentiation from epithelial cells is a specialized version of epithelial-mesenchymal transition (EMT), a physiological process in which epithelial cells can acquire the invasive and motile properties of mesenchymal cells [27]. Alveolar epithelial cells have been shown to undergo EMT in vivo during the development of pulmonary fibrosis [28]. There is also compelling evidence suggesting EMT in alveolar epithelial cells following exposure to TGF-*β* both *in vitro* and *in vivo* [29–31]. In addition, circulating bone marrow-derived fibrocytes behave like mesenchymal stem cells and migrate into sites of lung injury and become myofibroblasts. These fibrocytes express type 1 collagen and *α*-SMA and contribute to lung fibrosis [32]. It is interesting to note that TGF-*β*1-induced EMT is reversed by fibroblast growth factor-1, through ERK phosphorylation and Smad2 dephosphorylation [33]. This implies that the therapeutic role of FGF-1 should be defined in IPF, as inhibiting TGF-*β* globally may have adverse effects on its role in tissue homeostasis.

## 6. Extracellular Matrix and IPF

There is now mounting evidence suggesting that extracellular matrix (ECM) is involved in both normal physiology as well as in wide variety of pathophysiological processes. One possible mechanism of pulmonary fibrosis is the disruption of ECM which enables cell-to-cell contact of epithelial cells with fibroblasts, leading to the epithelial cell induction of fibroblast- and myofibroblast-derived signalling molecules. The end process of EMT is the degradation of underlying extracellular matrix with the formation of additional mesenchymal cells. Epithelial cell injury causes the release of a wide variety of growth factors, chemokines and MMPs, notably MMP-2, MMP-3, and MMP-9, interleukins, and prostaglandins. Under the influence of these signalling molecules, epithelial cells, acting together with inflammatory cells, induce basement membrane disruption and focal degradation of type IV collagen and laminin [34].

## 7. Role of TGF-***β*** in IPF

Transforming growth factor beta (TGF-*β*) is a pleiotropic regulatory cytokine that is ubiquitously expressed by all cells and tissues within the body. TGF-*β*1, of the three highly homologous mammalian isoforms (TGF-*β*1-3), is thought to play a central mediator role in wound healing and fibrosis [35–37]. There is compelling evidence that TGF-*β*1 plays a pivotal role in pathophysiology of IPF. There is increasing evidence pointing to the profibrogenic effects of TGF-*β* both in animal models of IPF and in IPF patients. Induction of EMT in alveolar epithelial cells following TGF-*β*1 overexpression in triple transgenic mice and via adenoviral-mediated gene transfer induces severe pulmonary fibrosis. In contrast, *ex vivo* alveolar epithelial cells cultured on laminin/collagen mixtures undergo programmed cell death when exposed to active TGF-*β* [28].

## 8. Integrin-Mediated Activation of TGF-***β***

The integrins are a large family of transmembrane heterodimeric cell adhesion receptors which mediate cell-to-surface interactions either to the extracellular cellular matrix or to specific cell-surface receptors during cell-cell interactions [38, 39]. They are composed of *α* and *β* dimers in a noncovalent complex, comprising 24 combinations of 18 *α* and 8 *β* subunits. These receptors function as important regulators that control whole sets of cellular processes including cell spreading, retraction, migration, and proliferation. Integrins contain the specialized binding regions for physical attachment of cells to the ECM, and through intracellular domains they form connections to various components of the actin cytoskeleton and a wide variety of interconnecting signalling adaptors [40]. 

The cross talk between integrins and TGF-*β* signalling is of considerable interest in a wide variety of physiological and pathophysiological processes including fibrosis, cancer, and wound healing. The molecular interactions between TGF-*β* and integrins *α*V*β*6 and *α*V*β*8 are of particular interest in the pathogenetic mechanisms of pulmonary fibrogenesis. The *α*V*β*6 integrin-dependent activation of TGF-*β* requires the binding of the *β*6 cytoplasmic tail to the actin cytoskeleton [41]. This activation of TGF-*β* is highly dependent on the association of inactive (latent) forms of TGF-*β* and TGF-*β* binding protein-1 (LTBP-1) of the large latent complex with *α*V*β*6 integrin [42]. Furthermore, lysophosphatidic acid and stimulation of protease-activated receptor 1 have shown to activate *α*V*β*6-mediated activation of TGF-*β*1 via RhoA and are implicated in the pathogenensis of acute lung injury and pulmonary fibrosis [43, 44]. Partial inhibition of *α*V*β*6 integrin-mediated activation of TGF-*β*1 using small molecule inhibitors has been shown to possess antifibrotic activity, without pulmonary inflammation and emphysema [45].

## 9. Role of IGFBP-5 in IPF

IGFBP-5 is one of the members of insulin-like growth-factor-binding protein (IGFBP) family which bind to and modulate the biological actions of insulin-like growth factors by interference with receptor binding although these IGFBPs also have IGF-independent actions [46]. Increased expression of IGFBP-5 has been described in fibrosis [47, 48]. Increased expression of IGFBP-5 at mRNA and protein levels was also evident *in vitro* in primary fibroblasts cultured from disease-affected skin of patients with systemic sclerosis/scleroderma, compared with the skin from their healthy twins. It has been demonstrated that IGFBP-5 induces collagen and fibronectin production from fibroblasts and induces fibroblast/myofibroblast transdifferentiation *in vitro* and *in vivo * [25, 26]. Finally, *in vivo* overexpression of IGFBP5, using replication-deficient adenovirus, induced skin fibrosis in mice which included increased thickness of the dermis and increased collagen bundle thickness [25] and induced pulmonary fibrosis with myofibroblastic changes [26]. Increased expression of *α*-SMA and vimentin in dermal fibroblasts was also evident with overexpression of IGFBP-5. Collectively, these findings suggest that upregulated expression of IGFBP5 could be an initiating event in ECM production and indicate its involvement in the development of fibrosis.

## 10. Integrin-Dependent Interaction of IGFBP-5

Integrin *α*V*β*6 expression is constitutively expressed at low levels in epithelial tissues and was significantly increased during injury but was unable to potentiate fibrosis [49]. Recently, we demonstrated that *α*V*β*6 integrin is also essential in addition to *α*2 and *β*1 integrins for the IGFBP-5-induced cell to extracellular matrix adhesion in MCF-7 cells. Using biosensor technology, we showed a novel, direct, high affinity interaction of IGFBP-5 with *α*2 and *β*1 integrins leading to the activation of ILK and Akt and improved epithelial cell adhesion and survival (our unpublished observations). Apoptosis of epithelial cells induces IGFBP-5 which activates tissue plasminogen activator (tPA), generating plasmin, and also interacts with several matricellular proteins [50]. Thus, IGFBP-5 may act as a central mediator in the initial response of epithelial cell injury. 

This provides an intriguing possibility in which IGFBP-5 acts indirectly, extracellularly, in a similar fashion to connective tissue growth factor (CTGF), which also serves as a mediator of the actions of TGF-*β*1 [51]. CTGF is structurally related to IGFBP-5 and interacts with integrins to elicit its actions, rather than using classic cell-surface receptors. In fact, there are similarities between IGFBP-5 and other members of the secreted cysteine-rich (CCN) protein family which includes CTGF, CYR61, and Nov. The CCN molecules were temporarily renamed IGFBP8-10 because of their structural relationship with the IGFBP family. However, we believe that IGFBP-5 may be a member of the CCN family since it binds and activates integrins, interacts with the ECM via a heparin-binding domain, and influences growth factor actions (IGFs) indirectly by binding to them. In analogous fashion, CTGF as well as binding to integrins and the ECM binds to VEGF [52] to inhibit its actions and bind to, and enhances the actions of, TGF-*β*1 [53]. 

**Figure 1:**
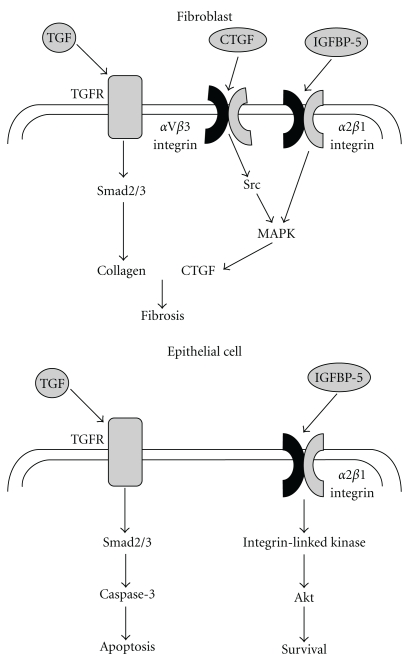
Differential effects of TGF-*β*1 and IGFBP-5 on mesenchymal and epithelial cells. In fibroblasts, TGF-*β*1 activates the Smad pathway resulting in activation of genes associated with fibrosis such as collagen, fibronectin, and CTGF. CTGF in turn further activates this process via an aVb3 integrin-dependent mechanism. IGFBP-5 binds to and activates *α*2*β*1 integrins and is capable of activating MAPK pathways in similar fashion to CTGF. In contrast, TGF-*β*1 activation of the Smad pathway results in an apoptotic pathway in epithelial cells, whereas IGFBP-5, again acting through *α*2*β*1 integrin, stimulates an adhesive, prosurvival pathway. Thus, IGFBP-5 enhances TGF-*β*1 actions in the mesenchymal compartment but inhibits them in the epithelium.

The adhesive responses to IGFBP-5 could be anticipated to enhance re-epithelialisation during injury by increasing the surface area of epithelial cells when epithelia are exposed to a mesenchymal environment, whilst concurrently inhibiting their migration into the mesenchymal compartment. Thus, IGFBP-5 would be anticipated to display antagonistic actions to TGF-*β*1 in the epithelial compartment whilst enhancing its actions in the mesenchymal compartment (Figure 1).

## 11. TGF-***β*** and IGFBP-5: Redundancy or Complementarity of Actions?

The recent studies implicating IGFBP-5 in the development of fibrosis begs the question “Do TGF-*β*1 and IGFBP-5 serve similar roles and, if so, why? We believe that, although their roles in the mesenchymal compartment are similar and certainly complementary, their actions in the epithelial compartment are different. Whereas TGF-*β*1 is clearly apoptotic and induces EMT in a proportion of the epithelial cells, thus disrupting the epithelial compartment and its functions both as a physical barrier and in, for example, gas exchange in the lung, the actions of IGFBP-5 are quite distinct. We have shown that IGFBP-5 induces an adhesive action [50] in epithelial cells exposed to a mesenchymal environment, leading to increased cell spreading and decreased migration and propose that this serves as a mechanism to prevent epithelial egress from, and mesenchymal ingress into, the epithelial compartment. IGFBP-5 also increases epithelial production of the basement membrane protein, laminin [54]. These actions would be anticipated to limit the boundary of the fibrotic response to the underlying stroma. Thus, IGFBP-5 may be a more relevant target in therapies of wound repair than TGF-*β*1, since it enhances fibrosis but limits scar formation to the mesenchymal compartment, thereby preventing excessive disruption of the epithelium. This contrasts with the uncontrolled fibrotic response characteristically produced by TGF-*β*1.

## 12. Clinical Implications

Fibrotic diseases in general, and idiopathic pulmonary fibrosis in particular, represent disease states for which no therapies currently exist. Targeting the major fibrotic agents may provide the prospect of developing such therapies, and TGF-*β*1 is, indeed, the target of research programmes seeking pharmacological inhibitors. The complementary roles of TGF-*β*1 and IGFBP-5 in alveolar epithelial cell injury provide additional options for drug targets although a better understanding of their precise mechanisms of action will be essential to enhance the prospects of success. Nevertheless, the potential protective role of IGFBP-5 in the epithelium is particularly amenable to aerosol-mediated drug delivery systems, since this would provide direct access to the site of action required for effective re-epithelialisation to occur. Possible approaches could include smaller domains of the IGFBP-5 molecule, which can exert these effects, drugs which mimic its adhesive, survival effects, or drugs, such as retinoic acid and vitamin D analogues, that are known to stimulate its production in epithelial cells [55–57].
